# Investigation of circRNA-miRNA-mRNA network in colorectal cancer using an integrative bioinformatics approach 

**Published:** 2021

**Authors:** Sepideh Kadkhoda, Farzaneh Darbeheshti, Nima Rezaei, Ghasem Azizi-Tabesh, Faezeh Zolfaghari, Sadollah Tavakolibazaz, Reza Taslimi, Javad Tavakkoly-Bazzaz

**Affiliations:** 1 *Department of Medical Genetics, School of Medicine, Tehran University of Medical Sciences, Tehran, Iran*; 2 *Breast Cancer Association (BrCA), Universal Scientific Education and Research Network (USERN), Tehran, Iran*; 3 *Research Center for Immunodeficiencies, Children's Medical Center, Tehran University of Medical Sciences, Tehran, Iran*; 4 *Department of Immunology, School of Medicine, Tehran University of Medical Sciences, Tehran, Iran*; 5 *Network of Immunity in Infection, Malignancy and Autoimmunity (NIIMA), Universal Scientific Education and Research Network (USERN), Tehran, Iran*; 6 *Department of Medical Genetics, School of Medicine, Shahid Beheshti University of Medical Sciences, Tehran, Iran*; 7 *Genomic Research Center, Shahid Beheshti University of Medical Sciences, Tehran, Iran*; 8 *Department of Gastrointestinal Dis., Imam Reza Hospital, Mazandaran University of Medical Sciences, Amol, Iran*; 9 *Department of Gastroenterology, Imam Khomeini Hospital, Tehran University of Medical Sciences, Tehran, Iran*; * *Sepideh Kadkhoda and Farzaneh Darbeheshti contributed equally to this study as first authors*

**Keywords:** circRNA, miRNA, Colorectal cancer, Microarray, Bioinformatics

## Abstract

**Aim:**

The aim of this study was to integrate both coding and non-coding available microarray data in the development of colorectal cancer (CRC) with bioinformatics analyses to attain a more inclusive pathobiologic map of their molecular interactions and functions.

**Background:**

Identification of competing endogenous RNAs (ceRNAs), especially circRNAs, has become a new hotspot in cancer research, although their roles and underlying mechanisms in CRC development remain mostly unknown.

**Methods:**

Microarray data was retrieved from the Gene Expression Omnibus (GEO) database and analyzed. Several bioinformatics tools and databases were applied for further elucidation. Principal component analysis (PCA) was run separately for four datasets. The dysregulated circRNA-miRNA-mRNA, co-expression, and protein-protein interaction (PPI) networks were established.

**Results:**

PCA discloses colorectal tumors; normal tissue can be distinguished not only by mRNAs expression profile, but also by both circRNA and miRNA expression profiles. In this study, 14 DE mRNAs, 85 DE miRNAs, and 36 DE circRNAs were identified in CRC tissue and compared with normal tissue. Taking their potential interactions into account, a circRNA-miRNA-mRNA network was constructed. The results disclosed some DE circRNAs with potential oncogenic (circ_0014879) or tumor suppressive (circ_0001666 and circ_0000977) effects. Finally, the PPI network suggests pivotal roles for DOCK2 and PTPRC dysregulation in the progression of CRC, possibly by facilitating tumor escape from immune surveillance.

**Conclusion:**

The current study proposes a novel regulatory network consisting of DE circRNAs, miRNAs, and mRNAs in CRC development that highlights the roles of DE circRNAs at the upstream of oncotranscriptomic cascade in CRC development, suggesting their potential to be utilized as both prognostic and therapeutic biomarkers.

## Introduction

 Colorectal cancer (CRC) was classified as the third most prevalent and second most lethal malignancy in 2018, regardless of patient gender (1). However, it can be well treated and managed with suitable and early diagnosis. Thus, a better understanding of molecular signatures, potential biomarkers, and therapeutic targets in CRC is necessary to gradually improve the diagnosis and treatment efficacy.

**Table 1:** Characteristics of four colorectal cancer-related GEO microarray datasets; T: tumor; N: normal

GEO	Platform	Year	Country	Sample size
GSE41657	GPL6480	2015	China	25 T/12 N
GSE128449	GPL4133	2019	Spain	31 T/5 N
GSE128449	GPL14767	2019	Spain	18 T/4 N
GSE126095	GPL19978	2019	China	10 T/10 N

**Table 2:** Characteristics of differentially expressed circRNAs in GSE126095 with log fold change ≥|3| which are involved in dysregulated circRNA-miRNA-mRNA network (Fig. 4A). The red and black rows show down-expressed and up-expressed circRNAs, respectively

circRNA	Alias	Log FC	Category	Chromosome	Host gene
hsa_circRNA_102619	hsa_circ_0000977	-5.2	exonic	chr2	NOL10
hsa_circRNA_104270	hsa_circ_0001666	-4.2	exonic	chr6	FAM120B
hsa_circRNA_104475	hsa_circ_0082182	3.6	exonic	chr7	FAM71F2
hsa_circRNA_100367	hsa_circ_0014879	3.3	exonic	chr1	DCAF8
hsa_circRNA_104640	hsa_circ_0001806	3.6	exonic	chr8	CSPP1
hsa_circRNA_103188	hsa_circ_0062682	3.4	exonic	chr22	TPST2
hsa_circRNA_103348	hsa_circ_0065214	3.2	exonic	chr3	SCAP
hsa_circRNA_101555	hsa_circ_0001955	4.1	exonic	chr15	CSNK1G1
hsa_circRNA_001846	hsa_circ_0000520	3.3	intragenic	chr14	RPPH1
hsa_circRNA_104499	hsa_circ_0082564	3.3	exonic	chr7	CREB3L2
hsa_circRNA_101744	hsa_circ_0005699	3.2	exonic	chr16	C16orf62
hsa_circRNA_100146	hsa_circ_0011385	3.9	exonic	chr1	EIF3I
hsa_circRNA_101145	hsa_circ_0028198	3.4	exonic	chr12	ANAPC7
hsa_circRNA_100053	hsa_circ_0009910	3	exonic	chr1	MFN2
hsa_circRNA_101164	hsa_circ_0028602	3.1	exonic	chr12	RNFT2
hsa_circRNA_102771	hsa_circ_0055377	3.1	exonic	chr2	CTNNA2

Today, high throughput technologies have aided the discovery and investigation of both coding and non-coding dysregulated transcripts in tumor cells. The pivotal roles of non-coding RNAs in gene expression regulation and, consequently, cancer initiation and progression have been determined. Recently, circular RNAs (circRNAs), as a newfound category of non-coding RNAs, were highlighted in cancer research. In relevant studies, circRNAs showed the potential to be diagnostic and prognostic biomarkers due to their high stability and tissue/stage specificity (2). In 2011, the competing endogenous RNA (ceRNA) hypothesis was proposed by Salmena et al. (3), who demonstrated that RNA transcripts communicate with each other through microRNA response elements (MREs). According to this hypothesis, the role of circRNA as a microRNA sponge and the consequent dysregulation of protein-coding gene expression have been established in different malignancies (4, 5). Although various circRNAs have been reported to participate in the pathogenesis of CRC, their molecular interactions with other transcripts are still unknown (6). These interactions could improve current knowledge about the underlying molecular mechanisms and potential targeted therapies in CRC. Integrating experimental data with bioinformatics could present a powerful method to disclose molecular interactions among different transcripts in tumor cells as well as their potential functions. 

The aim of this study was to integrate available microarray data concerning circRNAs, miRNAs, and mRNAs in CRC with bioinformatics analyses by consensus strategy to gain a more accurate comprehension of their molecular interactions and functions. A dysregulated circRNA-miRNA-mRNA network was constructed in colorectal tumors as well as co-expression and protein-protein interaction (PPI) networks. The findings propose a novel regulatory network underlying CRC progression and uncover molecular interactions between dysregulated transcripts in colorectal tumors. 

## Methods

**Differentially Expressed Genes from GEO**

To investigate and integrate differentially expressed (DE) RNAs in colorectal cancer, four microarray datasets, including both coding and non-coding transcripts, were selected from the Gene Expression Omnibus (GEO) database and analyzed between colorectal tumors and non-tumor tissue.

**Supplementary Figure 1:**
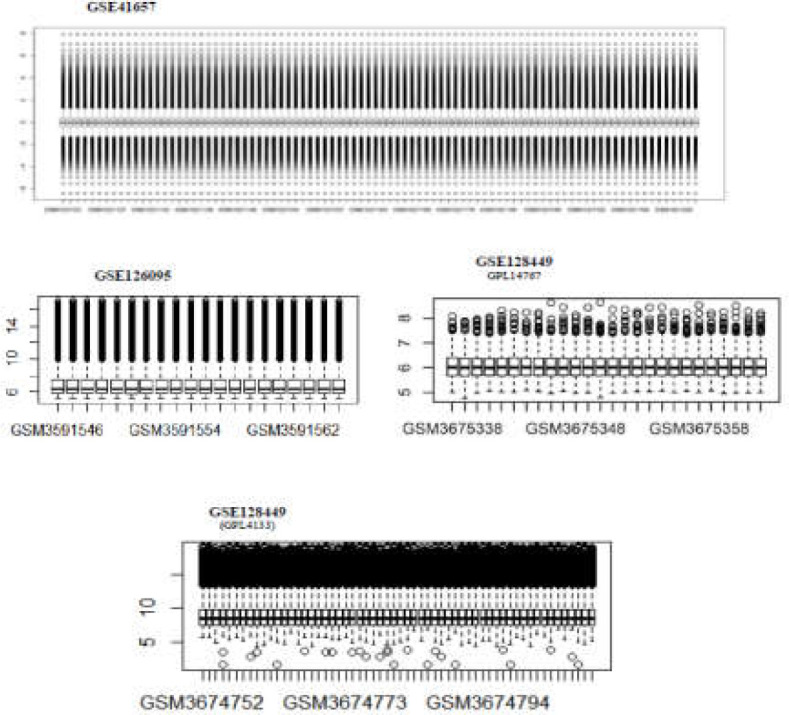
Boxplots of normalized data

Information about the four used datasets is summarized in Table 1. All raw expression data was normalized and log2-transformed (Supplementary Fig. 1). Limma, a Bioconductor package for the differential analysis of microarray data, was run to determine DE RNAs in each dataset with the following criteria: Two datasets, GSE41657 (platform GPL6480) and GSE128435 (platform GPL4133), were analyzed separately with a fold change ≥|2| and adj.p.value < 0.05 set as the cut-off point for the selection of DE mRNA. For this step, the following two criteria were used to define consensus: 1) A transcript has to be differentially expressed in the both experiments; 2) The change in expression in a transcript has to be in the same direction (up- or downregulation) in both experiments. Concerning the GSE41657 dataset, only adenocarcinoma and normal samples were extracted for analysis. The subseries 128435 is part of superseries GSE128449. 

GSE128449 (platform GPL14767), which is part of superseries GSE128449, was analyzed to determine DE miRNAs. In this step, DE miRNAs with a cut-off point of log fold change ≥|3| and adj.p.value < 0.05 were selected for further analysis.

**Figure 1:**
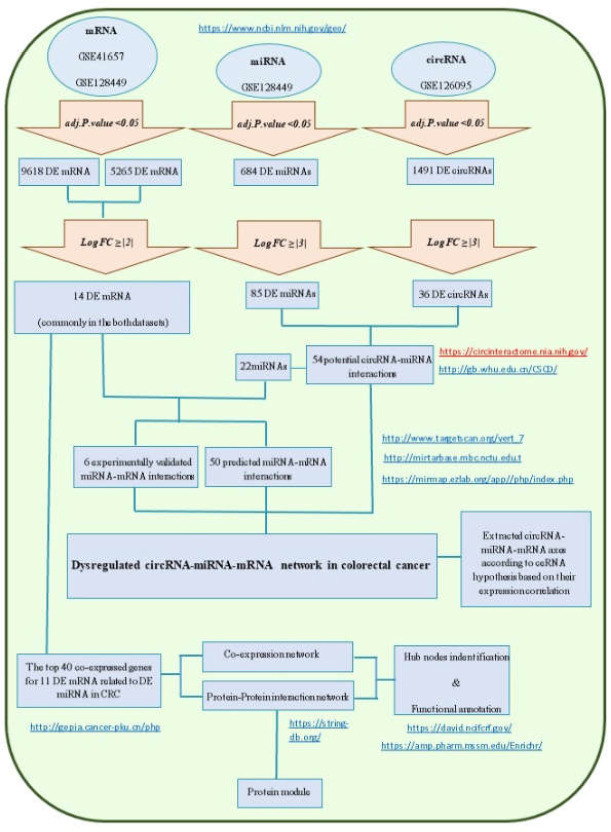
Flowchart of data and bioinformatics analyses

To assess DE circRNA in CRC, GSE126095 (platform GPL19978) was used, and transcripts with a cut-off point of adj.p.value < 0.05 and log fold change ≥|3| were retrieved. The flowchart of data and bioinformatics analyses is presented in Fig. 1. To investigate similarities and dissimilarities between samples, clustered heatmaps for the top 100 DE RNAs in the four datasets were analyzed.

**Principal component analysis (PCA)**

The PCA plot evaluates differences and similarities among samples and determines whether samples can be grouped. Hence, it can separate normal and tumor tissues based on their gene expression profiles. Scatterplots of PCA from four microarray data were generated separately using the ggfortify package in R.

**circRNA-miRNA-mRNA network**

First, the CircInteractome online database (7) was used to bioinformatically find potential miRNAs with a complementary seed region on DE ciRNAs. For each DE circRNA, all predicted miRNAs were obtained. Next, miRNAs that overlapped with predicted and DE miRNAs were gathered. After that, common targets of DE miRNAs with DE mRNAs that were retrieved from both GSE41657 and GSE128435 were assessed. To this end, miRTarBase (8) was used to detect experimentally validated miRNA-mRNA interactions. Also, two databases, TagetScan (9) and miRmap (10), were run for bioinformatics analysis of the predicted miRNA-mRNA interactions. It should be noted that only interactions that are commonly predicted in both databases were selected. Finally, a dysregulated circRNA-miRNA-mRNA network in colorectal cancer was constructed by Cytoscape version 3.6.1. Characteristics of the 16 DE circRNAs that are involved in this network are shown in Table 2. 

Then, the axes that showed expression status according to ceRNA hypothesis were extracted. In this step, it was presumed that circRNAs show opposite expression direction to their downstream miRNAs and same direction to downstream mRNAs in a circRNA-miRNA-mRNA axis. The Cancer-Specific CircRNA (CSCD) database (11) was used to visualize basic structural patterns of the circRNAs involved in the circRNA-miRNA-gene axis.

**Co-expression Network Construction**

The top 40 co-expressed genes in colorectal cancer for each DE mRNA were retrieved from the GEPIA database (12) according to the Cancer Genome Atlas (TCGA) data. The co-expression network was generated by Cytoscape, and hub nodes were identified according to the degree and betweenness centrality. Finally, the hub genes in this network were functionally analyzed by the DAVID database (13).

**Protein-Protein Interaction (PPI) Network Construction**

First, PPIs were identified by the String database (14), and then the PPI network was visualized by Cytoscape. To identify hub nodes, degree and betweenness centrality were considered. Next, MCODE, a Cytoscape app, was run to find the most significant protein module. Then, the Enrichr database (15) was used to understand the biological function of hub genes in the PPI network. 

**Figure 2:**
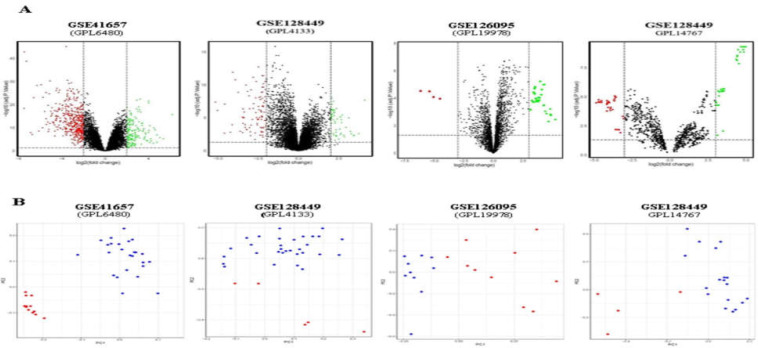
**A:** The Volcano plot of significant differentially expressed mRNAs (GSE41657 and GSE128449,GPL4133) with ≥ |2| log fold change, circRNAs (GSE126095) and miRNAs (GSE128449,GPL14767) with ≥ |3| log fold change in colorectal tumors and normal colorectal tissues. Red and green dots show down and up regulated RNAs, respectively. **B:** The scatterplot of PCA from the gene expression profiles in tumor and normal colorectal tissuses, including two mRNA microarray datasets (GSE41657 and GSE128449,GPL4133), one circRNA microarray dataset (GSE126095), and one miRNA microarray dataset (GSE128449,GPL14767). The blue points represent colorectal tumor cells, whereas the red ones show normal cells. Distribution of information with respect to differential expression between tumor and normal tissues

**Table 3:** Functional annotation of hub genes in co-expression network by DAVID database

Hub genes	GO terms
GAS7	Cell cycle arrest, Regulation of transcription, DNA-templated, Nervous system development, Cell differentiation
C10ORF54	Single organismal cell-cell adhesion
TSPAN1	Cell proliferation, Cell migration, Cell surface receptor signaling pathway, Positive regulation of endocytosis, Protein stabilization
RELL1	Plasma membrane, Microtubule cytoskeleton, Integral component of membrane

**Oncogenomic and Oncotranscriptomic Analysis**

cBioPortal v.3 (16) was run to identify somatic alterations of hub genes in 526 colorectal tumor samples according to TCGA, PanCancer atlas. In addition, GEPIA was used to assess the expression of hub genes in CRC tissue compared with normal tissue based on TCGA data. 

## Results

**Differentially Expressed mRNAs, miRNAs, and circRNAs**

To obtain differentially expressed (DE) protein coding genes in CRC, two GEO datasets (GSE 41657 and GSE 128435) were analyzed. In this step, genes with adj.p.value < 0.05 and log fold change ≥| 2 | were selected. Subsequently, 14 common DE genes were found in both datasets that showed the same dysregulation direction (Supplementary Table 1). Two of these genes are up-expressed and 12 are down-expressed. Details of the analysis are shown in Fig. 1.

A GEO dataset concerning the expression of miRNAs in CRC (GSE128449) was analyzed. In this step, 85 DE miRNAs with cutoff criteria of adj.p.value < 0.05 and log fold change ≥| 3 | were obtained (Supplementary Table 2).

As mentioned, GSE126095 was examined to find DE circRNAs in CRC. Thirty-six DE circRNAs with adj.p.value < 0.05 and log fold change ≥| 3 | were obtained (Supplementary Table 3). 

**Figure 3:**
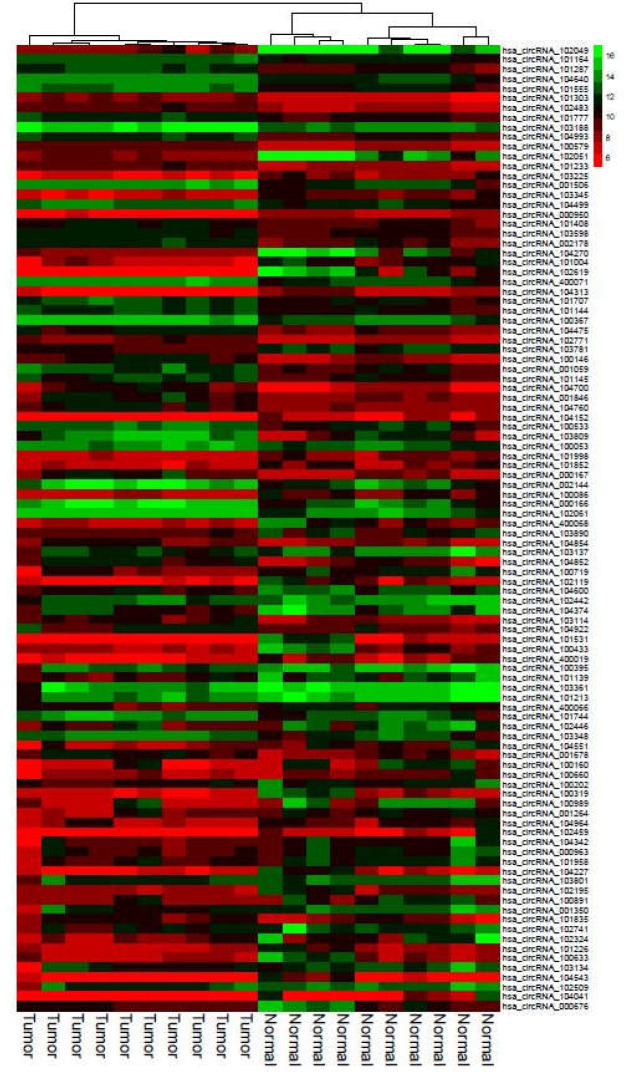
Clustered heatmap for the top 100 differentially expressed circRNAs according to GSE126095. Rows represent circRNAs and columns represent tissue types

**Supplementary Figure 2:**
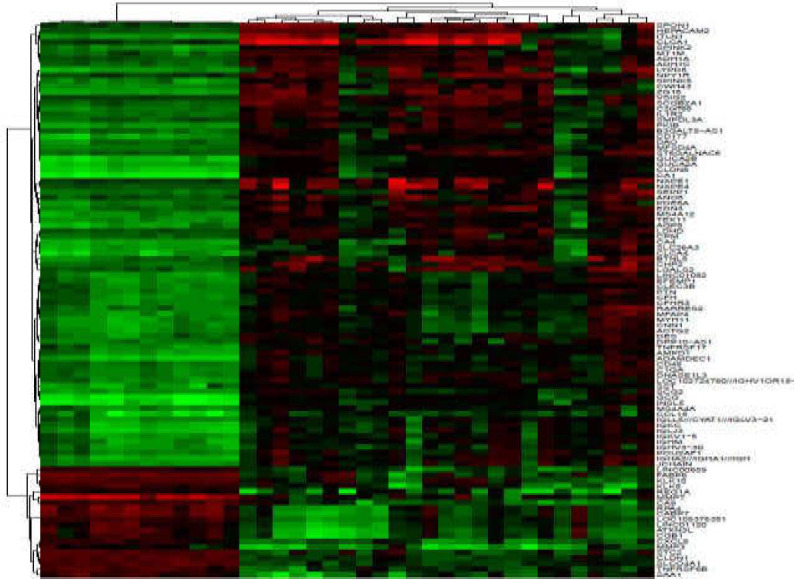
Clustered heatmap for the top 100 differentially expressed mRNAs according to GSE41657. Rows represent circRNAs and columns represent tissue types

**Supplementary Figure 3:**
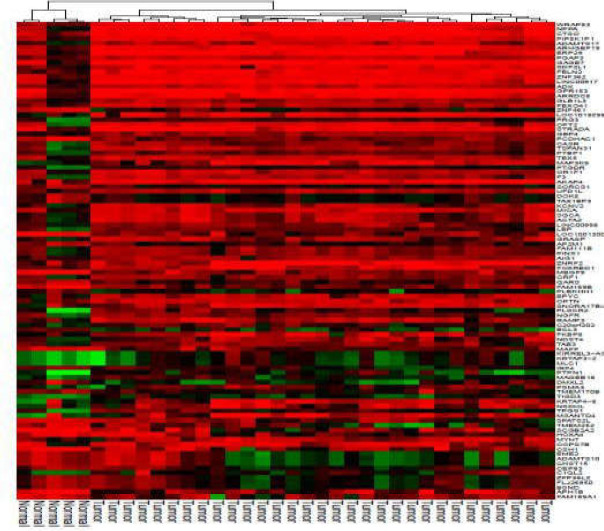
Clustered heatmap for the top 100 differentially expressed mRNAs according to GSE128449. Rows represent circRNAs and columns represent tissue types

**Supplementary Figure 4:**
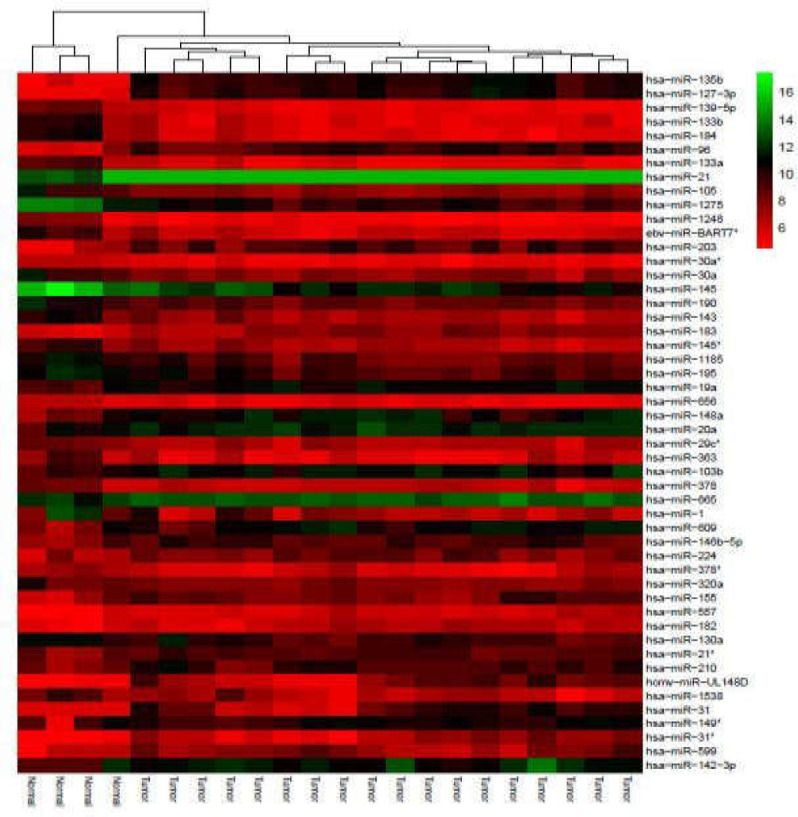
Clustered heatmap for the top 50 differentially expressed circRNAs according to GSE128449. Rows represent circRNAs and columns represent tissue types

Clustered heatmaps for all analyzed datasets are shown in Fig. 3 and Supplementary Figures 2-4. In addition, volcano plots of DE RNAs in the four datasets have been drawn (Fig. 2A).

The gene expression PCA plot provides insight into the association between samples. As shown in Fig. 2B, colorectal tumor and normal tissues could be distinguished not only by mRNA expression profiles, but also by both circRNA and miRNA expression profiles.

**Potential DE circRNA-DE miRNA and DE miRNA-mRNA interactions**

According to the circInteractome database, 54 DE circRNA-DE miRNA interactions are bioinformatically predicted. Three databases were run to investigate DE gene-DE miRNA interactions. Six experimentally validated interactions by mirTarbase and 50 bioinformatically predicted interactions by TargetScan and miRmap were consistently obtained (Supplementary Table 4).

**CircRNA- miRNA- mRNA network**

**Figure 4:**
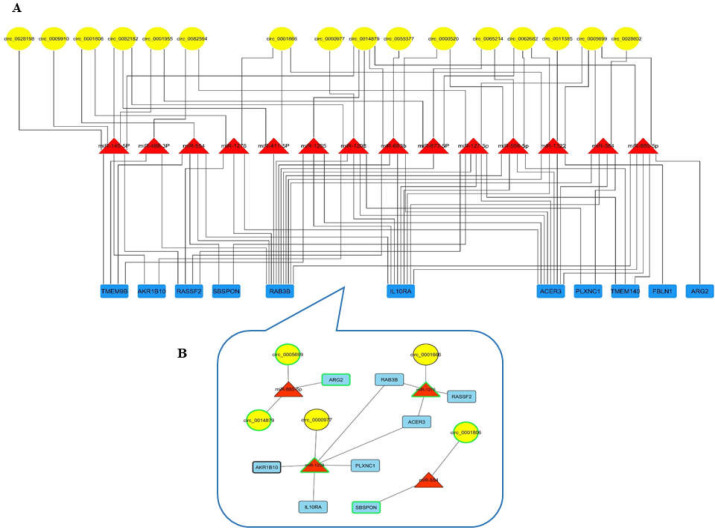
**A:** Dysregulated circRNA-miRNA-mRNA network in CRC. The yellow ellipses present circRNAs, the red triangles present miRNAs and the blue rectangles present mRNAs. The network was constructed by Cytoscape. **B: **Extracted axes from circRNA-miRNA-mRNA network that have an expression correlation according to ceRNA hypothesis. The nodes with black borderline are down-expressed and the nodes with green borderline are up-expressed in analyzed GEO datasets. The yellow ellipses present circRNAs, the red triangles present miRNAs and the blue rectangles present mRNAs

**Figure 5:**
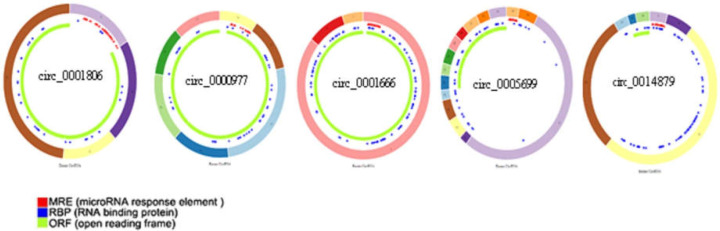
Structural patterns of the six circRNAs involved in circRNA-miRNA-gene axis (ceRNA hypothesis). Figures were retrieved from Cancer-Specific CircRNA (CSCD).

A dysregulated circRNA- miRNA- mRNA network in colorectal cancer was constructed with 16 circRNAs, 14 miRNAs and 11 mRNAs (Fig. 4A). According to the ceRNA hypothesis, the extracted axes, which consist of five circRNAs, four miRNAs, and eight mRNAs, are presented in Fig. 4B. The circRNAs involved in these axes are available in the Cancer-Specific CircRNA (CSCD) database (Fig. 5).

**Co-expression network construction**

**Figure 6:**
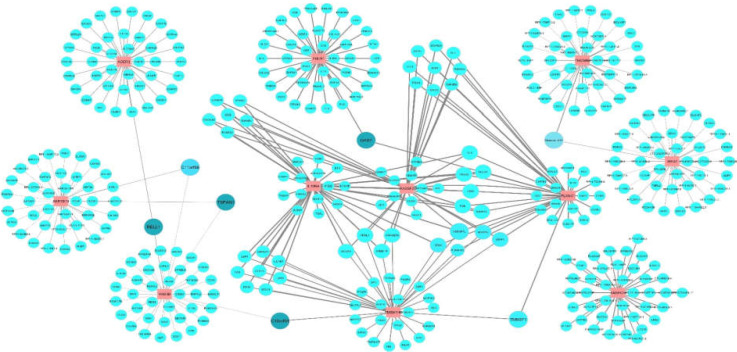
Co-expression network of 11 differentially expressed (DE) genes in colorectal cancer. The top 40 co-expressed gene for each DE gene were achived from GEPIA webserver based on TCGA data. Data are visualized by Cytoscape. The nodes in red present 11 DE genes. The blue nodes present co-expressed genes, their size changes according to the node degree and the node color changes gradually from light blue to dark blue according to the betweenness centrality. The width of the edge indicates Pearson Correlation Coefficient (PCC).

As mentioned, 11 dysregulated genes related to DE miRNAs were detected. Their top 40 co-expressed genes in CRC were used to construct a co-expression network (Fig. 6). In this network, GAS7, C10ORF54, RELL1, and TSPAN1 were considered as hub genes because of their higher degree and betweenness centrality. In addition, functional analysis of hub genes indicates their roles in cancer-related processes, such as cell proliferation and migration (Table 3).

**Protein-Protein interaction (PPI) network**

**Figure 7.**
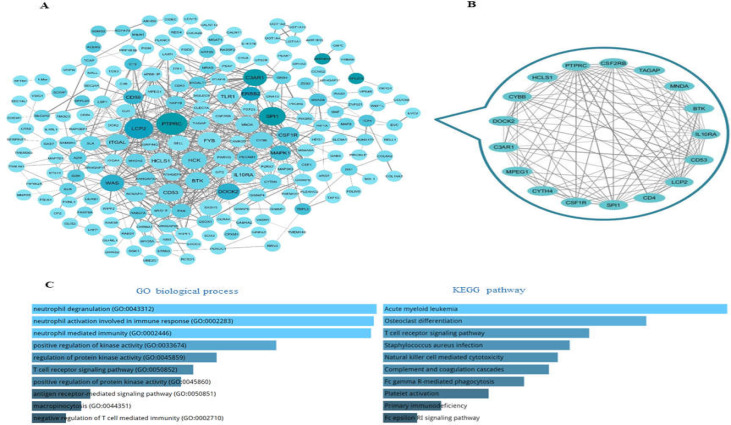
**A:** Protein-Protein Interaction (PPI) network. Data were retrieved from String based on 11 differentially expressed (DE) genes and their co-expressed genes in CRC and visualized by Cytoscape. The color of nodes were determined based on betweenness centrality and their size based on degree; darker color and bigger nodes have more betweenness centrality and degree. Also for determining of the edges width, combined score was considered. **B:** The main module is extracted from PPI by MCODE cytoscape app. **C:** Functional annotation for the five hub nodes including SPI1, DOCK2, PTPRC, C3AR1, and LCP2. The graphs were achieved from Enrichr and sorted based on combined score. The longer bar and brighter color represent term that is more

The PPI network of 11 DE genes and their co-expressed genes was established after unconnected nodes 

were removed based on String output (Fig. 7A). This network shows PTPRC, SPI1, C3AR1, LCP2, and DOCK2 genes as hub nodes. MCODE was run to detect significant modules (Fig. 7B). It should be noted that all hub nodes are involved in the detected module. 

Functional annotation according to the Enrichr web server revealed the roles of hub genes in the immune system (Fig. 7C).

**Oncotranscriptomic and Oncogenomic Analysis**

**Table 4:** Summary of previous studies on dysregulation of circRNAs and miRNAs involved in circRNAs/miRNAs/mRNAs axes (Fig. 3) in different cancer types. The red and black transcripts present down- and up-expression in CRC, respectively, based on our analyses

Transcript	Article	Dysregulation	Cancer type
circRNAs			
hsa_circ_0000977	(Guo, Fesler et al., 2018) (37)	up	Pancreatic cancer
hsa_circ_0001666	(Hou, Tan et al., 2018) (38)	down	Thyroid cancer
hsa_circ_0014879	(Su, Lin et al., 2016) (39)	up	Esophageal cancer
	(Qiu, Wang et al., 2019) (40)	up	Hepatocellular carcinoma
hsa_circ_0005699	(Ou, Lin et al., 2017) (41)	up	Hepatocellular carcinoma
	(Guan, Ma et al., 2019) (4)	down	Gastric cancer
hsa_circ_0001806	(Li, Liu et al., 2019) (42)	up	Ovarian cancer
	(Qiu, Wang et al., 2019) (40)	up	Hepatocellular carcinoma
miRNAs			
miR-885-5p	(Reid, Sokolova et al., 2012) (43)	down	Colorectal cancer
	(Afanasyeva, Mestdagh et al., 2011) (44)	down	Neuroblastoma
	(Zhang, Yin et al., 2016) (45)	down	Hepatocellular carcinoma
miR-554	(Chen, Li et al., 2013) (46)	down	Breast cancer
	(Jin, Dai et al., 2016) (47)	down	Glioma
miR-1276	(Xiong, Dang et al., 2018) (48)	up	Hepatocellular carcinoma
	(Hou, Jian et al., 2015) (49)	up	Pancreatic cancer
miR-1208	(Anauate, Leal et al., 2019) (50)	up	Gastric cancer
	(Huppi, Volfovsky et al., 2008) (51)	down	Breast cancer
	(Huppi, Volfovsky et al., 2008) (51)	down	Colorectal cancer

**Figure 8:**
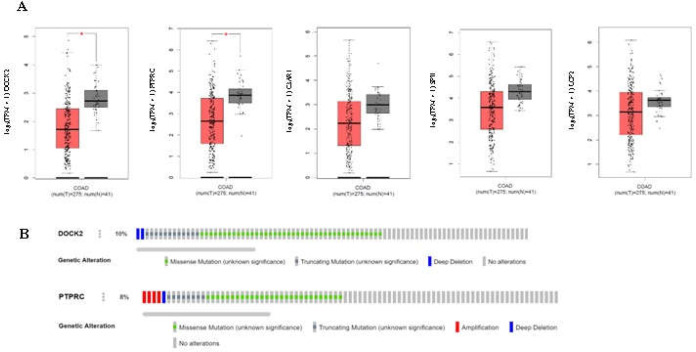
**A:** Expression analysis of five hub genes of PPI network between tumors and normal samples. The boxplots were retrieved from GEPIA based on TCGA data including expression of DOCK2, PTPRC, C3AR1, SPI1, and LCP2 from left to right, respectively. Tumors and normal samples present in red and gray, respectively. Red star indicates significant difference between tumors and normal tissues (P.value ≤0.01). **B:** Genetic alterations of DOCK2 and PTPRC in 526 colorectal tumor samples (the two hubgenes with significant difference expression between tumors and normal tissues). Figures were extracted from cBioPortal based on TCGA, PanCancer atlas

Expression analysis for five hub nodes of the PPI network using GEPIA revealed that while all five are down-expressed in tumors compared with normal tissue, only the dysregulation of DOCK2 and PTPRC is statistically significant (Fig. 8A).

Investigation of DOCK2 and PTPRC somatic mutations in colorectal cancer revealed a fairly high frequency (10% and 8%, respectively) of genetic alterations in tumors. As shown in Fig. 8B, the main proportion of these alterations is composed of missense and truncating mutations.

## Discussion

Investigation of non-coding RNAs as well as identification of competing endogenous RNAs (ceRNAs) have become new hotspots in cancer research. 

Among them, circRNAs are more prominent due to their specific characterizations, such as high stability, time- and tissue-specificity. circRNAs can play an important role in gene expression regulation at both transcriptional and posttranscriptional levels (17). Various studies have demonstrated that dysregulated circRNAs are involved in cancer initiation and progression. For example, up-regulation of hsa_circ_0000069 and its role in cell proliferation, migration, and invasion were observed in CRC (18). Recent evidence has disclosed that circRNAs could sponge miRNA and suppress their functions. Nonetheless, few studies have been conducted concerning circRNA molecular interactions in CRC.

miRNAs are a class of small noncoding RNAs which have pivotal roles in regulating gene expression at the post-transcriptional level by suppressing the target mRNA. It is well known that dysregulated miRNAs are involved in several oncopathways and result in cancer development. In this study, 36 DE circRNAs and 85 DE miRNA with log fold change ≥ |3| were identified in CRC tissue compared with non-tumor tissue as well as 14 DE mRNAs, which consistently showed dysregulation in both GSE41657 and GSE128435 GEO datasets. These DE transcripts were integrated using several databases and bioinformatics tools in order to construct a dysregulated circRNA-miRNA-mRNA network in CRC. In addition, mRNA, miRNA, and circRNA datasets were analyzed separately to assess their ability to separate colorectal tumor and normal samples. The results revealed that each of them alone could distinguish between colorectal tumors and normal samples.

According to the ceRNA hypothesis, it was presumed that circRNAs, as miRNA sponges, show opposite expression direction to their downstream miRNAs and same direction to downstream mRNAs in a circRNA-miRNA-mRNA axis, as 11 axes with this expression status were extracted from the circRNA-miRNA-mRNA network, including five circRNAs, four miRNAs, and eight mRNAs. Previous cancer research concerning these transcripts is summarized in Table 4. However, most of these circRNAs and miRNAs have not been previously reported in CRC. It should be noted that six of the seven detected circRNAs are available in the Cancer-Specific CircRNA Database (CSCD), and the location of their microRNA response elements (MRE) are shown in Fig. 4.

The current study established the potential dysregulated circRNA-miRNA-mRNA axes that could play pivotal roles in CRC oncotranscriptomic. The present analyses revealed that circRNAs can show both oncogenic (oncoCirc) and tumor suppressive functions in CRC. For example, circ_0014879, an up-expressed exonic circRNA, could be an oncoCirc through circ_0014879/miR-885-5p/ARG2 axis. miR-885-5p is bioinformatically predicted to be sponged by circ_0014879. Interestingly, miR-885-5p is a tumor suppressor miRNA and down-expressed in different cancers (Table 4) as well as in the current results. ARG2 is an enzyme that plays a part in the immunosuppressive tumor microenvironment and tumorigenesis. ARG2 is predicted to be a miR-885-5p target. This protein shows overexpression and association with poor prognosis in different cancers. In 2019, Youjun Wu et al. demonstrated that expression of ARG2 is significantly higher in CRC samples than in normal tissue (19). In addition, they revealed that the downregulation of ARG2 inhibits the growth of colorectal cancer cells. According to the current investigation, ARG2 is significantly up-expressed in both GSE41657 and GSE128435 datasets with log fold changes of 2.15 and 2.05, respectively. Therefore, it is suggested that overexpression of circ_0014879 as an oncoCirc could result in the upregulation of the ARG2 oncogene through inhibition of the miR-885-5p function in CRC.

On the other hand, circ_0001666, a down-expressed exonic circRNA, could be a tumor suppressive circRNA in CRC through sponging miR-1276 in the circ_0001666/miR-1276/RASSF2 axis. It was revealed that RASSF2 inhibits tumor cell growth, suggesting that it is a tumor-suppressor gene in CRC (20). Moreover, the present results disclose the potential tumor suppressive roles of circ_0000977 in CRC through sponging miR-1208. Interestingly, this miRNA might post-transcriptionally inhibit two tumor suppressor genes, i.e. IL10RA and AKR1B10 in CRC. In 2018, Zadka et al. reported that IL10RA is down-expressed in CRC tissue and shows a negative correlation with clinical stage and proliferation (21). It was shown that AKR1B10 expression is significantly decreased in CRC samples, and its down-expression correlates with decreased survival and poor prognosis of patients (22). In addition, AKR1B10 knock-down resulted in the inhibition of apoptosis in colorectal cancer cells. It should be noted that the expression of RASSF2, IL10RA and AKR1B10 was down-regulated in both GSE41657 and GSE128435 datasets with a log fold change < -2. Collectively, it can be proposed that under-expression of both circ_0001666 and circ_0000977, as tumor suppressive circRNAs, contributes to the down-regulation of some tumor suppressive genes in CRC, including RASSF2, IL10RA, and AKR1B10. These findings should be validated through further functional studies in the future.

**Supplementary Figure 5:**
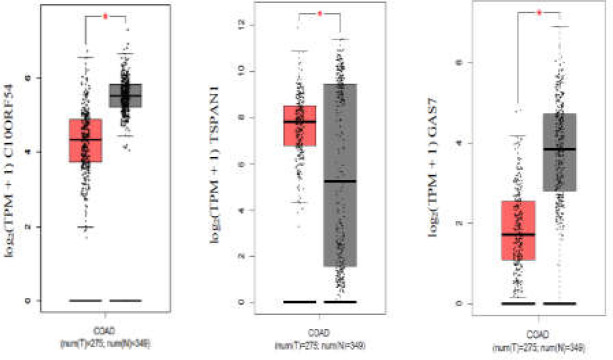
Expression analysis of C10ORF54, TSPAN1 and GAS7 between tumor s and normal samples. The boxplots were retrieved from GEPIA based on TCGA data. Tumors and normal samples present in red and gray respectively. Red star indicates significant difference betwee n tumors and adjacent normal tissues (P.value ≤0.01).

In the next step, we constructed a dysregulated co-expression network in CRC, including 11 DE genes and their co-expressed genes. This network shows GAS7, C10ORF54, TSPAN1, and RELL1 genes are hub nodes and could play important roles in the progression of CRC tumors as far as the expression profile is concerned. Functional annotation based on the DAVID database revealed their roles in cell proliferation, cell-cell adhesion, cell migration, and cell differentiation (Table 3). Consistently, previous studies have demonstrated their dysregulation in CRC. Remarkable down-regulation of GAS7 due to promoter hypermethylation was reported in CRC (23, 24). It is suggested that the downregulation of GAS7 results in high metastatic ability of tumor cells. RELL1 is a family member of tumor necrosis factor receptor (TNFR) that induces apoptosis (25, 26). This study proposed the important role of RELL1 in CRC oncotranscriptome for the first time; according to the current results, RELL1 shows a significant expression correlation with two down-regulated DE genes, namely RAB3B and ACER3, in CRC-related TCGA data. C10ORF54 (VISTA) is a protein-coding gene which codes immunoregulatory receptors. This protein inhibits the T-cell response and acts during adaptive immune responses (27). Its expression can be a predictive biomarker as far as cancer immunotherapies are concerned (28). Interestingly, the analyses of the current study based on TCGA data (Supplementary Fig. 5) showed that GAS7 and C10ORF54 are significantly downregulated in CRC tissue. However, in 2018, Shan Xie et al. reported that C10ORF54 expression was significantly high in CRC tissue compared with normal tissue (29). It seems that this controversy should be noted in CRC patients who are supposed to receive immunotherapies. Another hub gene in the co-expression network is TSPAN1 which functions in cell mitosis and differentiation. TCGA data showed the significant up-expression of TSPAN1 in CRC tumor compared with normal tissue (Supplementary Fig. 5). It has been reported that overexpression of TSPAN1 is correlated with poor prognostic factors in CRC patients (30). In 2010, Chen et al. reported that the suppression of TSPAN1 results in reduced proliferation and invasion of colon cancer cells (31).

To assess the protein interactions between DE mRNAs and their co-expressed genes, the current study established a PPI network using the STRING search tool. This network revealed two hub nodes, DOCK2 and PTPRC, which significantly downregulate in CRC tumors according to TCGA data (Fig. 7A). Oncogenomic analysis showed that a notable proportion of their downregulation is probably attributable to truncating mutations (Fig. 7B), although it seems that transcriptional and post-translational regulatory mechanisms are involved in the down-regulation of DOCK2 and PTPRC2 in CRC. Hematopoietic cells express DOCK2 which modulates the activation and migration of immune cells. DOCK2 has been shown to be involved in inflammatory diseases such as malignancies (32). Yu et al. demonstrated that DOCK2 mutation has a high frequency (7.7%) in colon tumors, and low expression of DOCK2 is associated with poor prognoses for patients (33). Another investigation has documented that DOCK2 is a prominent hypermethylated gene in CRC tissue (34). The PTPRC protein is necessary for T-cell activation, and its mutation is associated with severe combined immunodeficiency. Down-regulation of PTPRC has been reported in several cancer types (35, 36). The current study showed that DOCK2 and PTPRC are not only hub nodes of dysregulated PPI in CRC based on degree and betweenness centrality, but are also involved in the module identified with the MCODE algorithm (Fig. 6B). Functional enrichment analysis of module members indicated their roles in the immune system (Fig. 6C). Taken together, it is suggested that the downregulation of DOCK2 and PTPRC in CRC contributes to the escape of tumors from immune surveillance; accordingly, their dysregulation could be involved in resistance to immunotherapy.

In conclusion, this study applied the combination of both coding and non-coding microarray data as well as bioinformatics tools to construct the dysregulated circRNA-miRNA-mRNA network and detect ceRNA axes in CRC. Potential oncoCirc (circ_0014879) and tumor suppressive circRNAs (circ_0001666 and circ_0000977) in CRC were identified that could become both prognostic and therapeutic biomarkers. In addition, the PPI network implies the pivotal roles of immune system-related proteins such as DOCK2 and PTPRC in CRC progression, which could be important in immunotherapies.

**Supplementary table 1:** 

Gene.symbol	Gene.title
SBSPON	somatomedin B and thrombospondin type 1 domain containing
ARG2	arginase 2
ACER3	alkaline ceramidase 3
TMEM9B	TMEM9 domain family member B
RAB3B	RAB3B, member RAS oncogene family
FBLN1	fibulin 1
PLXNC1	plexin C1
IL10RA	interleukin 10 receptor subunit alpha
AKR1B10	aldo-keto reductase family 1 member B10
RASSF2	Ras association domain family member 2
TMEM140	transmembrane protein 140
IL1R2	interleukin 1 receptor type 2
APOE	apolipoprotein E
NRAP	nebulin related anchoring protein
